# Undiagnosed Pheochromocytoma During Pregnancy Presenting Initially With Neurological Manifestations: A Case Report

**DOI:** 10.1155/carm/5345705

**Published:** 2026-08-24

**Authors:** Tahereh Babaei, Seyed Ahmad Hosseini, Hadi Majidi, Somayeh Sheidaei, Nazanin Nosrati, Azadeh Nazari Tileh Noei, Kosar Babaei

**Affiliations:** ^1^ Deputy of Treatment, Mazandaran University of Medical Sciences, Sari, Iran, mazums.ac.ir; ^2^ Cardiovascular Research Center, Rajaie Cardiovascular Institute, Tehran, Iran; ^3^ Clinical Research Development Unit of Imam Khomeini Hospital, Mazandaran University of Medical Sciences, Sari, Iran, mazums.ac.ir; ^4^ Department of Radiology and Nuclear Medicine, School of Medicine, Mazandaran University of Medical Sciences, Sari, Iran, mazums.ac.ir; ^5^ Department of Pathology, Faculty of Medicine, Mazandaran University of Medical Sciences, Sari, Mazandaran, Iran, mazums.ac.ir; ^6^ Department of Emergency Medicine, Khatam Al-Anbia Hospital, Mazandaran University of Medical Sciences, Behshahr, Iran, mazums.ac.ir; ^7^ Students Research Committee, Neyshabur University of Medical Sciences, Neyshabur, Iran, neyshabur.ac.ir

**Keywords:** adrenal hemorrhage, case report, pheochromocytoma, pregnancy, transverse myelitis

## Abstract

A 50‐year‐old pregnant woman (G3Ab2) who had her fourth IVF procedure during the first trimester of her pregnancy is described in this article as having a rare incidence of acute paraplegia. The patient’s early symptoms included headache, sudden lower limb weakness, urine retention, back discomfort, and uncontrolled hypertension. The patient had a history of infertility, thyroidectomy, hypertension, and repeated spontaneous abortions. After transverse myelitis was confirmed by spinal MRI, methylprednisolone pulse therapy and plasmapheresis were started as treatments. Subsequent analysis of CSF fluid showed purulent meningitis. An unexpected abortion occurred 48 h later. She had fast hypotension, confusion, and a hemoglobin drop from 10 to 5 g/dL over 72 h. A tumor in the left adrenal gland and a sizable, active hematoma in the right adrenal gland were discovered by abdominal imaging. After a right adrenalectomy and mass draining were done in an emergency, the pheochromocytoma was finally diagnosed. The patient was briefly stabilized postsurgery but ultimately succumbed. This case is one of the limited instances documented in the scientific literature, when the emergence of neurological symptoms from transverse myelitis serves as the initial manifestation of pheochromocytoma, in conjunction with high‐risk pregnancy, IVF, and an aggressive clinical trajectory. Consequently, in instances of unstable or early hypertension in pregnancy with neurological symptoms, differential diagnosis should include neuroendocrine tumors such as pheochromocytoma.

## 1. Introduction

Pheochromocytoma is an uncommon neuroendocrine tumor that can manifest during pregnancy, with an estimated incidence of about 1 in 50,000 pregnancies [1]. The management of this tumor during pregnancy is rare and presents considerable hazards to both the mother and the fetus [1]. Pheochromocytoma is frequently misdiagnosed during pregnancy due to its low prevalence and clinical similarities with gestational hypertension [2]. A delayed or missing diagnosis markedly deteriorates maternal and fetal outcomes. In instances of severe hypertension before 20 weeks of gestation, particularly when associated with variable blood pressure, intermittent headaches, palpitations, or diaphoresis, pheochromocytoma should be included in the differential diagnoses [3]. Timely identification and suitable intervention can decrease maternal and fetal mortality rates from above 50% to below 5% and 15%, respectively [3]. The 5‐year survival rate for benign pheochromocytoma varies from 84% to 96%, but it decreases to around 40% in malignant cases. Malignancy constitutes approximately 10% of cases, with prevalent metastatic sites being the lymph nodes, liver, and lungs, but spinal involvement is regarded as uncommon [4]. Pheochromocytoma predominantly arises from the adrenal medulla (85%) and, to a lesser extent, from the sympathetic ganglia (15%), leading to excess catecholamine secretion and a wide array of clinical manifestations [5]. Approximately 20% of pregnancy cases remain undetected due to vague clinical manifestations and diagnostic constraints [5]. Notwithstanding advancements in maternal healthcare, hypertensive disorders during pregnancy remain inadequately investigated, with a predominant clinical focus on preeclampsia, thus marginalizing additional significant illnesses such as pheochromocytoma in diagnostic frameworks [6]. A multitude of variables contribute to this elevated death rate, including the restricted application of radiologic and nuclear imaging in pregnancy, the infrequency of the tumor, and its diverse clinical manifestations [7]. Moreover, anatomical and physiological alterations, including uterine enlargement and heightened intra‐abdominal pressure, may trigger tumor activation or hemorrhage, thereby complicating its care [8–10]. Clinicians must recognize the distinct diagnostic and treatment concerns of pheochromocytoma during pregnancy [11]. Establishing explicit criteria for assessment in pregnant women with chronic or newly developed hypertension is crucial for prompt diagnosis and care. Pheochromocytoma, originating from chromaffin cells of the adrenal medulla or sympathetic ganglia, constitutes 0.2%–0.4% of secondary hypertension diseases and may induce life‐threatening hypertensive crises [12, 13]. Moreover, the traditional triad of headache, palpitations, and diaphoresis may be less discernible in pregnant individuals, rendering identification more challenging [14]. This case report intends to emphasize an uncommon yet significant diagnostic situation, given the rarity of pheochromocytoma initially manifesting with acute neurological symptoms such as transverse myelitis during pregnancy, necessitating prompt detection and multidisciplinary care.

## 2. Case Description

**FIGURE 1:**
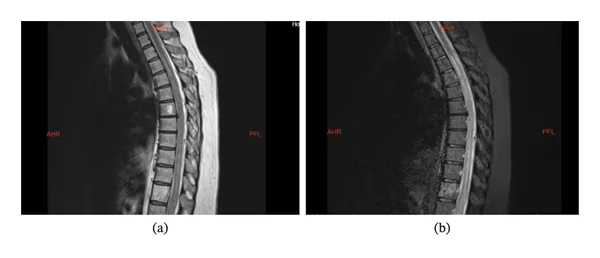
(a) Spinal cord from T3–conus medullaris has a heterogeneous signal and diffuse central cord edema, which is consistent with transverse myelitis. (b) T10 vertebral body hemangioma is noted.

A 50‐year‐old gravida three pregnant woman who had two previous spontaneous abortions (G3Ab2) showed up in the first trimester after her fourth attempt at in vitro fertilization (IVF). Chronic hypertension, proteinuria, a complete thyroidectomy 23 years prior because of papillary thyroid carcinoma, and repeated miscarriages were among her notable medical history. She complained of a severe headache that started suddenly, uncontrolled hypertension, bilateral lower limb weakness, paresthesia, urine retention, and lower back and coccygeal pain when she was admitted. Complete paraplegia was found by neurological testing, with both lower limbs showing a motor strength score of 0/5. Assessments of the cranial nerve and upper limbs showed no abnormalities. As part of the initial evaluation for secondary causes of hypertension, abdominal ultrasonography was performed, showing normal kidneys. Doppler ultrasonography of the renal arteries was also unremarkable, with no evidence of renovascular disease. No adrenal abnormality was identified on the initial examination. Transverse myelitis was confirmed by magnetic resonance imaging (MRI) of the cervical, thoracic, and lumbosacral spine, which revealed diffuse central cord edema extending from T3 to the conus medullaris and heterogeneous signal (Figure 1a).

**FIGURE 2:**
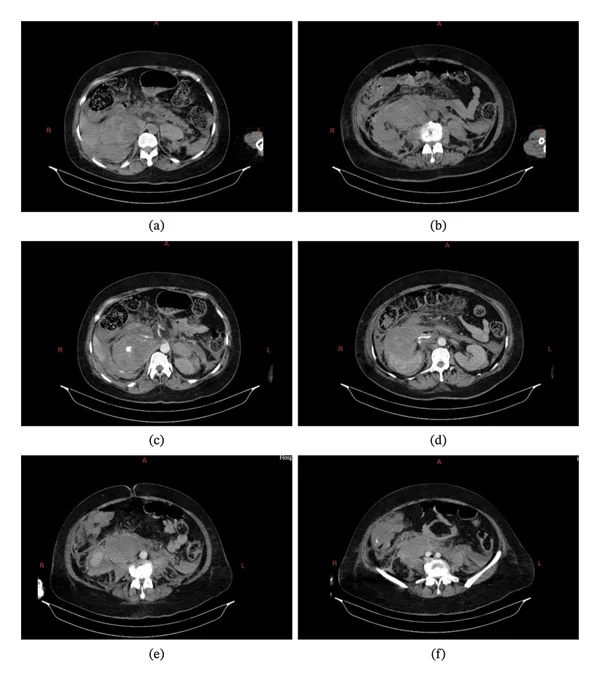
(a, b) Abdominopelvic CT scan shows a left adrenal mass and a massive hematoma of the right adrenal. (c–f) A left adrenal mass and a right adrenal hematoma with extravasation from intravascular to intrahematoma, presenting active bleeding.

Additionally, a hemangioma was seen at the T10 vertebral body (Figure 1b). To rule out autoimmune, rheumatologic, and viral causes, numerous laboratory tests were conducted (Table 1). Empirical treatment with high‐dose intravenous methylprednisolone and plasmapheresis was initiated due to the suspected diagnosis of autoimmune transverse myelitis. Autoimmune and rheumatologic investigations, including ANA, ANCA, antiphospholipid antibodies, anti‐dsDNA, complement levels, and viral PCR assays, were unremarkable. The autoimmune and infectious work‐up for transverse myelitis was negative, except for inflammatory CSF findings. After a lumbar puncture, cerebrospinal fluid (CSF) analysis revealed purulent bacterial meningitis. CSF smear and culture showed no gross abnormalities; however, based on the overall CSF findings, broad‐spectrum intravenous antibiotics were initiated, and methylprednisolone was discontinued (Table 2). At the same time, methyldopa and hydralazine were used as part of antihypertensive medication to control blood pressure. The patient’s sudden abortion occurred 48 h later. She experienced rapid hypotension, disorientation, and a sharp decline in hemoglobin from 10 to 5 g/dL throughout the next 72 h. A 40 × 25‐mm hemorrhagic lesion in the right adrenal gland with an increasing retroperitoneal hematoma and a left‐sided adrenal mass were discovered by abdominal–pelvic ultrasonography and computed tomography (CT) (Figure 2a–f).

**TABLE 1:** The patient’s serological, viral, and autoimmune lab data.

Parameter (full name)	Result	Unit
Wright agglutination test	Negative	—
2‐Mercaptoethanol (2 ME) test	Negative	—
Coombs–Wright test	Negative	—
Hepatitis C virus antibody (HCV Ab)	0.3	Index
Human immunodeficiency virus‐1/2 p24 antigen (HIV‐1/2 p24 Ag)	0.4	Index
Hepatitis B surface antigen (HBsAg)	0.2	Index
Interferon‐gamma release assay (QuantiFERON‐TB Gold, IGRA)	Negative	—
Antinuclear antibody (ANA)	1:40 (Negative)	Titer
Antiphospholipid IgG antibody	2.4 (Negative)	U/mL
Complement Component 3 (C3)	84	mg/dL
Complement Component 4 (C4)	19.4	mg/dL
Antidouble‐stranded DNA antibody (anti‐dsDNA)	1.7	IU/mL
Anticardiolipin IgG antibody	1.5	GPL‐U/mL
Anticardiolipin IgM antibody	1.6	MPL‐U/mL
Lupus anticoagulant (LA)	42.2	Seconds
Anti‐β2 glycoprotein I IgG antibody	1.5	U/mL
Anti‐β2 glycoprotein I IgM antibody	1.4	U/mL
Anti‐Ro (SSA) antibody	0.3	U/mL
Anti‐La (SSB) antibody	1.8	U/mL
Cytoplasmic antineutrophil cytoplasmic antibody (c‐ANCA)	< 1:10	Titer
Perinuclear antineutrophil cytoplasmic antibody (p‐ANCA)	< 1:10	Titer
Total hemolytic complement activity (CH50)	70	U/mL

**TABLE 2:** Cerebrospinal fluid (CSF) analysis results.

Parameter (full name)	Result	Unit
Red blood cell count (RBC)	8784	cells/μL
White blood cell count (WBC)	2700	cells/μL
Neutrophils	95%	%
Lymphocytes	5%	%
Wright agglutination test (CSF)	Negative	—
Cerebrospinal fluid glucose	23	mg/dL
Cerebrospinal fluid total protein	394.5 (high)	mg/dL
Cerebrospinal fluid albumin	37 (high)	mg/dL
CSF IgG Index	0.01 (low)	—
CSF oligoclonal bands (OCB)	Negative	—
Cerebrospinal fluid angiotensin‐converting enzyme (ACE)	< 0.05	U/L
Cerebrospinal fluid immunoglobulin G (IgG)	0.5 (low)	mg/dL
Total nucleated cells (CSF)	9072	cells/μL
CSF Gram stain and bacterial culture	No organisms detected/no growth	—
Varicella‐zoster virus (VZV) DNA PCR	Undetected	—
Cytomegalovirus (CMV) DNA PCR	Undetected	—
Herpes simplex virus (HSV) DNA PCR	Undetected	—

**FIGURE 3:**
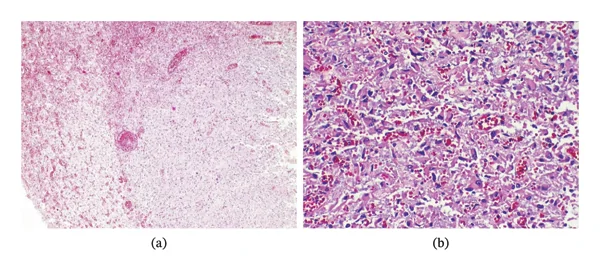
(a) Histopathological staining with hematoxylin and eosin (H&E) at 100x magnification. The adrenal tumor reveals a nested pattern of cells (Zellballen) with a hemorrhagic area. (b) The H&E staining of the tumor at 400x magnification demonstrates polygonal cells with abundant granular cytoplasm and round to oval nuclei with mild pleomorphism. A vascular stroma surrounds the cells.

Continuous bleeding was suggested by evidence of active contrast extravasation into the hematoma. The right adrenalectomy and excision of the retroperitoneal tumor were done during an emergency laparotomy. Histopathological analysis validated the pheochromocytoma diagnosis. The microscopic examination showed a conventional nested (Zellballen) architecture composed of polygonal tumor cells with round‐to‐oval nuclei and abundant granular cytoplasm, all set within a vascular stroma. Large areas of bleeding were observed, consistent with the tumor’s vascular fragility (Figure 3a,b). The neurological symptoms may have been caused by excessive catecholamine release, extreme blood pressure fluctuations, and subsequent spinal ischemia, even though transverse myelitis is an unusual initial presentation for pheochromocytoma. This atypical overlap demonstrates the difficulty of determining what is happening in such circumstances. After the surgery, the patient was stable for a short time, but then her blood pressure dropped, her heart rate slowed, and she may have had multiorgan failure. The patient received comprehensive intensive care, including inotropic support, vasopressor therapy, and mechanical ventilation. Despite maximal medical management and aggressive supportive care, the patient developed refractory multiorgan failure and ultimately died (Figure 3).

## 3. Discussion

This report describes a rare and complex case of acute transverse myelitis complicated by bacterial meningitis in early pregnancy, resulting in a fatal outcome. Ten percent of these tumors are malignant. This case highlights the importance of appropriate preoperative medical optimization with *α*‐adrenergic blockade (e.g., phenoxybenzamine), which remains the cornerstone of perioperative blood pressure management in patients with pheochromocytoma. The absence of this intervention may have contributed to the patient’s unfavorable clinical course [4]. The clinical manifestations of pheochromocytoma are frequently nonspecific and ambiguous, potentially leading to confusion with conditions such as preeclampsia or other pregnancy‐related disorders. The classic triad of pheochromocytoma headache, palpitations, and diaphoresis is well recognized but is present in only a minority of patients. During pregnancy, these symptoms may be overlooked or attributed to other pregnancy‐related conditions or concurrent illnesses. Gestational hypertension often manifests during the 20th week of amenorrhea, although hypertension resulting from pheochromocytoma may arise at any point throughout pregnancy. A significant component of this case is the patient’s history of thyroidectomy, suggesting the potential presence of an undiscovered hereditary disease, such as Multiple Endocrine Neoplasia Type 2 (MEN2). The present case is noteworthy for combining several uncommon clinical features. Unlike most reported cases of pheochromocytoma during pregnancy, which typically involve a unilateral adrenal tumor, our patient had bilateral adrenal involvement, consisting of a hemorrhagic right adrenal pheochromocytoma and a contralateral adrenal mass. The diagnosis was substantially delayed because the patient lacked the classic catecholamine‐related symptoms, including episodic headache, palpitations, and diaphoresis. Instead, the initial presentation was dominated by acute neurological manifestations consistent with transverse myelitis, directing the diagnostic evaluation toward neurological and autoimmune disorders rather than an underlying endocrine etiology. This atypical clinical presentation highlights the importance of considering pheochromocytoma in pregnant women presenting with severe or labile hypertension before 20 weeks of gestation, particularly when accompanied by unexplained neurological deficits or rapid clinical deterioration. Although the findings from a single case cannot be generalized, this report broadens the clinical spectrum of pheochromocytoma during pregnancy. It may facilitate earlier diagnosis and timely multidisciplinary management in similar high‐risk clinical scenarios. This underscores the necessity for adequate long‐term monitoring in individuals with a history of medullary thyroid cancer or family disorders that predispose to pheochromocytoma [5]. Neurological symptoms may trigger a broad differential, including rare endocrine malignancies, and secondary causes of hypertension should be rigorously examined, especially in individuals with labile or early‐onset hypertension during pregnancy.

## 4. Conclusion

This report highlights the diagnostic difficulties of pheochromocytoma during pregnancy, particularly when the tumor presents with unusual neurological manifestations such as acute lower limb paralysis (paraplegia). Paroxysmal hypertension is often attributed to gestational hypertension in pregnant patients and is therefore undoubtedly largely overlooked as pheochromocytoma. The initial presentation of transverse myelitis in this patient, followed by a progressive and severe clinical course, hypotension, sudden abortion, and the identification of bilateral adrenal hematomas, highlights the clinical complexity of this disorder and the need for a rapid diagnostic and therapeutic response. Based on this experience, we recommend that pregnant women presenting before 20 weeks’ gestation with chronic or labile hypertension accompanied by severe headache, neurological deficits, or atypical clinical features should undergo early evaluation for secondary hypertension, including adrenal imaging and biochemical testing for pheochromocytoma whenever clinically feasible. Early multidisciplinary involvement of obstetricians, endocrinologists, neurologists, and surgeons may facilitate timely diagnosis and improve maternal–fetal outcomes. Early recognition and timely intervention are a key to improving clinical outcomes. This report emphasizes the need to consider pheochromocytoma as a possible differential diagnosis in pregnant women presenting with hypertensive crises accompanied by neurological symptoms, especially in patients with a history of endocrine disorders, infertility, or pregnancy at an advanced age.

## Funding

The authors received no financial support for the research, authorship, and/or publication of this article.

## Ethics Statement

This study was approved by the Ethics Committee of Mazandaran University of Medical Sciences (approval number: IR.MAZUMS.REC.1404.179). The procedures used in this study adhere to the tenets of the Declaration of Helsinki.

## Consent

Written informed consent was obtained from the patient for publication of this case report and any accompanying images.

## Conflicts of Interest

The authors declare no conflicts of interest.

## Data Availability

The data that support the findings of this study are available on request from the corresponding author. The data are not publicly available due to privacy or ethical restrictions.
